# Impact of [^177^Lu]Lu-PSMA-617 Radioligand Therapy on Reference Organ Uptake Assessed by [^68^Ga]Ga-PSMA-11-PET/CT

**DOI:** 10.3390/cancers15153878

**Published:** 2023-07-30

**Authors:** Daniel Groener, Jennifer Wichert, Magdalena Adams, Nicolai Mader, Konrad Klimek, Christina Nguyen Ngoc, Justus Baumgarten, Christian Happel, Philipp Mandel, Felix K. H. Chun, Nikolaos Tselis, Frank Grünwald, Amir Sabet

**Affiliations:** 1 Department of Nuclear Medicine, University Hospital Frankfurt, Theodor Stern Kai 7, 60590 Frankfurt, Germany; daniel.groener@kgu.de (D.G.); jennifer.wichert@t-online.de (J.W.); magdalena.adams@kgu.de (M.A.); nicolai.mader@kgu.de (N.M.); konrad.klimek@kgu.de (K.K.); christina.nguyenngoc@kgu.de (C.N.N.); justus.baumgarten@kgu.de (J.B.); christian.happel@kgu.de (C.H.);; 2 Department of Urology, University Hospital Frankfurt, Theodor Stern Kai 7, 60590 Frankfurt, Germany; philipp.mandel@kgu.de (P.M.); felix.chun@kgu.de (F.K.H.C.); 3 Department of Radiation Oncology, University Hospital Frankfurt, Theodor Stern Kai 7, 60590 Frankfurt, Germany; nikolaos.tselis@kgu.de

**Keywords:** PSMA, [^177^Lu]Lu-PSMA-617, [^68^Ga]Ga-PSMA-11 PET/CT, metastatic castration-resistant prostate cancer, reference organs

## Abstract

**Simple Summary:**

The transmembrane protein prostate-specific membrane antigen (PSMA) has emerged as a target for both molecular imaging and PSMA-directed radioligand therapy (RLT). Normal organs, including the liver and salivary glands, exhibit physiological PSMA-ligand accumulation and have, therefore, gained interest as decisive landmarks for the semiquantitative classification of tumoral uptake on [^68^Ga]Ga-PSMA-11 PET/CT imaging. The presented study aims to assess the change in uptake to reference organs, including the liver, parotid and salivary glands after radioligand therapy (RLT) with [^177^Lu]Lu-PSMA-617 in relation to pretreatment imaging metrics.

**Abstract:**

This study aims to assess the change in uptake to reference organs, including the liver, parotid and salivary glands after radioligand therapy (RLT) with [^177^Lu]Lu-PSMA-617 in relation to pretreatment imaging metrics. Eighty-five patients with mCRPC underwent [^68^Ga]Ga-PSMA-11 PET/CT imaging prior to (pre RLT PET) and after (post RLT PET) a median of 3 (IQR 2-6) RLT cycles with [^177^Lu]Lu-PSMA-617. PSMA-positive tumor burden was stratified into 4 groups based on modified PROMISE criteria (oligofocal, multifocal, disseminated, diffuse). Uptake (SUV_mean_, SUV_max_) in liver tissue, parotid and submandibular glands was measured. A control group was established with 54 patients who had received two separate PET acquisitions following the same protocol (PET1, PET2) within 12 months for localized or oligofocal prostate cancer without RLT in the interim. Baseline uptake values (SUV_mean_, SUV_max_) in parotid (10.8 ± 3.2, 16.8 ± 5.4) and submandibular glands (11.3 ± 2.8, 18.1 ± 4.7) are 2-fold compared to liver uptake (4.9 ± 1.4, 7.7 ± 2.0), with no significant change between PET 1 and PET 2 in the control group. In the RLT group, increasing tumor burden class is significantly associated with decreasing uptake in the liver (*p* = 0.013), parotid (*p* < 0.001) and submandibular glands (*p* < 0.001); this tumor sink effect by respective tumor burden is widely maintained after RLT (*p* = 0.011, *p* < 0.001, *p* < 0.001). RLT has a significant impact on salivary gland uptake with decreasing values per patient in all groups of disease burden change (up to −30.4% in submandibular glands, *p* < 0.001), while liver tissue shows rising values in patients with declining tumor burden throughout RLT (+18.6%, *p* = 0.020). Uptake in liver tissue and salivary glands on [^68^Ga]Ga-PSMA-11 PET/CT imaging is inversely related to tumor burden prior to and following RLT with [^177^Lu]Lu-PSMA-617. Per patient, salivary gland uptake is further reduced throughout RLT independently from tumor burden, while changes in liver uptake remain burden-dependent. Liver and salivary gland uptake-derived metrics and segmentation thresholds may thus be of limited value when used as reference for response assessment to RLT.

## 1. Introduction

Selective delivery of radionuclides to the type II transmembrane protein prostate-specific membrane antigen (PSMA) has been increasingly adopted as a potent concept for diagnostics and treatment of prostate cancer. Overexpressed on the surface of prostate cancer cells, PSMA provides a tumor-specific binding site for radioligands suited for both positron emission tomography (PET) imaging and radioligand therapy (RLT) [1]. The closely linked application of molecular imaging and therapy has been termed “theranostics”. Along this line of care, patients can be individually selected for cancer treatment based on the presence of a biomolecular target, and therapeutic success is monitored accordingly. The broader implementation of such theranostic concepts in clinical medicine is expected to promote personalized cancer treatments and to play a beneficial role, both from the patient perspective and potentially on a population-based level [2,3]. Recently, PSMA-directed theranostic concepts have also been investigated in the context of non-prostatic tumor entities [4,5]. Normal organs, including the liver and salivary glands, exhibit physiological PSMA-ligand accumulation and have, therefore, gained interest as decisive landmarks for semiquantitative classification of tumoral uptake. 

The VISION trial, an open-label, multicenter phase 3 study, yielded high anti-tumoral activity of [^177^Lu]Lu-PSMA-617 RLT, with low rates of organ toxicity in metastatic castration-resistant prostate cancer (mCRPC) [6]. Patient selection mandated tumor uptake to exceed the liver uptake in target lesions on pretherapeutic PET/CT assessment [7]. Liver uptake-derived metrics, such as the tumor-to-liver ratio (TLR), have also been investigated in the context of response assessment to RLT [8], and semi-automated segmentation algorithms frequently incorporate the liver uptake as threshold for quantification of total PSMA-positive tumor volume [9,10]. Recently, the PSMA PET tumor-to-salivary gland ratio (PSG) was suggested as a surrogate for the prognostication of treatment response [11]. 

Liver and salivary gland uptake values are also relied upon as landmarks for stratification of focal tumoral uptake in PET/CT reporting frameworks [12,13]. The updated PROMISE (V2) criteria for molecular imaging maintain a four-point scoring system for PSMA expression; based on the blood pool, liver and parotid gland uptake as thresholds, the E-PSMA reporting system defines visual scoring levels (V-Score) accordingly. 

Measurement of reference organ uptake on PET/CT imaging can be subject to considerable interpatient variability. Several studies have addressed a so-termed tumor sink effect by tracer sequestration to high-volume tumor tissue, resulting in a decrease in tracer uptake in non-tumoral tissue, including reference organs [14,15,16]. While the existence of tumor sink phenomena has been demonstrated in single timepoint cohorts, there remains a lack of understanding as to what extent uptake to reference organs is altered in patients undergoing RLT. Beta radiation from [^177^Lu]Lu-PSMA-617 radioligands may affect PSMA expression as well as organ vitality over the course of multiple RLT cycles, as previously shown, and may thus also have an impact on organ-based stratification schemes. 

This study aims to investigate the change in uptake to reference organs, including the liver and salivary glands (parotid and submandibular gland) on [^68^Ga]Ga-PSMA-11 imaging throughout the course of RLT with [^177^Lu]Lu-PSMA-617. Standardized uptake metrics (SUV_max_, SUV_mean_) are examined in relation to tumor burden and RLT treatment activity.

## 2. Materials and Methods

### 2.1. Patients

A total of 85 patients with mCPRC who underwent RLT with [^177^Lu]Lu-PSMA-617 and received [^68^Ga]Ga-PSMA-11 imaging at baseline, and following RLT were included in this retrospective single-center series (RLT group). Prior to RLT, indications were confirmed by a multidisciplinary team. Exclusion criteria for the analysis were: (1) other malignant diagnoses in past medical history, (2) any changes in androgen deprivation therapy (ADT) between the two scans, (3) suspected or proven metastases to the liver at any time, or (4) any relevant decline in liver function not attributable to extrahepatic tumor progression. Blood parameters to monitor liver function, including aspartate aminotransferase (AST), alanine aminotransferase (ALT), gamma-glutamyl transferase (GGT), bilirubin, and albumin were registered at baseline and after RLT. Common terminology criteria of adverse events (CTCAE) were applied to record relevant changes to blood values. 

A control group of 54 patients with diagnosed prostate cancer, who received two ^68^Ga-PSMA-11-PET/CT scans at least six months apart adhering to the same imaging routine, was evaluated for comparison (control group). Patients in the control group were mandated to have localized or low-volume disease, with less than six lesions in either of the performed scans and no systemic treatments before or between the two ^68^Ga-PSMA-11-PET/CT exams. Production and administration of [^68^Ga]Ga-PSMA-11 PET/CT and [^177^Lu]Lu-PSMA-617 were performed in accordance with the legal regulations set out in the German Drug Registration and Administration Act (AMG § 13 2b). All patients gave written consent prior to [^68^Ga]Ga-PSMA-PET/CT imaging and before each RLT cycle. Retrospective data analysis was approved by the ethics committee of Goethe University Frankfurt (approval number: 310/18).

### 2.2. Radiopharmaceutical Synthesis and PET/CT Imaging Procedure

^68^Gallium was eluted from a ^68^Ge/^68^Ga radionuclide generator (GalliaPharm, Eckert & Ziegler Radiopharma, Berlin, Germany), and the radiolabeling of PSMA-11 (ABX GmbH, Radeberg, Germany) followed an established protocol [13]. In the control group, the mean injected activity was 131 ± 18 MBq for the first scan (PET 1) and 134 ± 23 MBq for the second scan (PET 2), yielding no significant differences in comparison (*p* = 0.372). Image acquisition was initiated 63 ± 14 min (PET 1) and 63 ± 16 min (PET 2) after injection (*p* = 0.913), respectively. The median time interval between PET 1 and PET 2 was 12 (IQR 8–20) months. In the RLT group, baseline PET/CT (pre RLT PET) was performed with a mean injected activity of 131 ± 25 MBq;, the mean activity for post-RLT scans (post RLT PET) was 130 ± 26 MBq (*p* = 0.883). Injection-to-acquisition time was 64 ± 16 (pre RLT PET) and 66 ± 18 min (post RLT PET) (*p* = 0.636), respectively. Median time from the last treatment cycle to post RLT PET was 8 (IQR 6–12) weeks. Whole-body images were acquired from vertex to mid-thigh. A Biograph 6 PET/CT scanner (Siemens, Erlangen, Germany) was used, with decay, scatter, and attenuation correction performed in accordance with the joint EANM and SNMMI consensus statement [14]. 

### 2.3. PET/CT Imaging Assessment

An iterative ordered-subset expectation maximization (OSEM) algorithm was applied for reconstruction using 4 iterations and 8 subsets and gaussian filtering (Syngo TrueD, Vers 61.b, Siemens Healthcare, Erlangen, Germany). The matrix size was 168 × 168 with 5 mm slice thickness. A dedicated software package (OsiriX MD, Version 10.0.4, Pixmeo, Switzerland) was utilized for PET and CT data set fusion and further quantitative analysis. Mean standardized uptake in the liver (SUV_mean_ liver) was measured using a 30 mm spherical volume of interest (VOI) within the right liver lobe; the maximum standardized uptake value (SUV_max_) was taken from within this VOI. To quantify parotid and salivary gland uptake, SUV_max_ and SUV_mean_ were extracted from within a 40% isocontour. The right and left salivary glands were compared, yielding no significant side difference in tracer uptake (Supplement S1). Thus, for further analysis, the right salivary glands were included as surrogates for both sides, as also suggested by the PROMISE reporting framework [17]. Tumor burden was classified by consensus of two trained nuclear medicine physicians and based on modified PROMISE criteria (V2) [13]: (1) oligofocal (<6 lesions), (2) multifocal (6–20 lesions), (3) disseminated (≥20 lesions) disease, and (4) diffuse bone marrow involvement (miTNM, M1b, dmi). Change in tumor burden throughout RLT was assessed based on a recently met consensus [18]: a significant decline in tumor burden was defined as a reduction in uptake/tumor volume by >30%; unchanged tumor burden was defined as a change of uptake/tumor volume ≤ 30%; a significant increase in tumor burden was defined as an increase in uptake/tumor volume by >30% or the peripheral expansion of diffuse bone marrow involvement. 

### 2.4. 177. Lu-PSMA-617 Radiolabeling and Administration

Radiolabeling of PSMA-617 with ^177^LuCl_3_ was carried out as described previously [19,20]. [^177^Lu]Lu-PSMA-617 was administered by slow intravenous injection over 30–60 s, preceded and followed by infusion of 1000 mL of saline solution. RLT was performed as an in-patient procedure. Patients received a median of 3 (IQR 2–6) cycles of [^177^Lu]Lu-PSMA-617 RLT given at intervals of 4 to 8 weeks, with a mean cumulative treatment activity of 28.9 ± 20.0 GBq. 

### 2.5. Statistical Analysis

Statistical analyses were performed with SPSS (version 28.0, IBM, Armonk, NY, USA), and GraphPad Prism (version 9.5.1, GraphPad Software, San Diego, CA, USA) was used for plotting. Continuous variables are presented as median values with interquartile range (IQR) or mean ± standard deviation (SD). Categorical variables are reported as frequencies with percentages. A paired *t*-test was used for intraindividual analysis. For intergroup comparison of various categories of tumor burden, a Kruskal–Wallis test was utilized. All tests were two-sided, and *p*-values < 0.05 were considered statistically significant. Association of continuous parameters was analyzed using parametric correlation (Pearson’s correlation coefficient denoted with r). 

## 3. Results

Patient characteristics are detailed in Table 1. A total of 85 mCRPC patients with various extents of baseline tumor burden underwent RLT with [^177^Lu]Lu-PSMA-617. In all patients, liver, parotid and submandibular gland uptake could be quantified in both PET/CT acquisitions.

**Figure 1:**
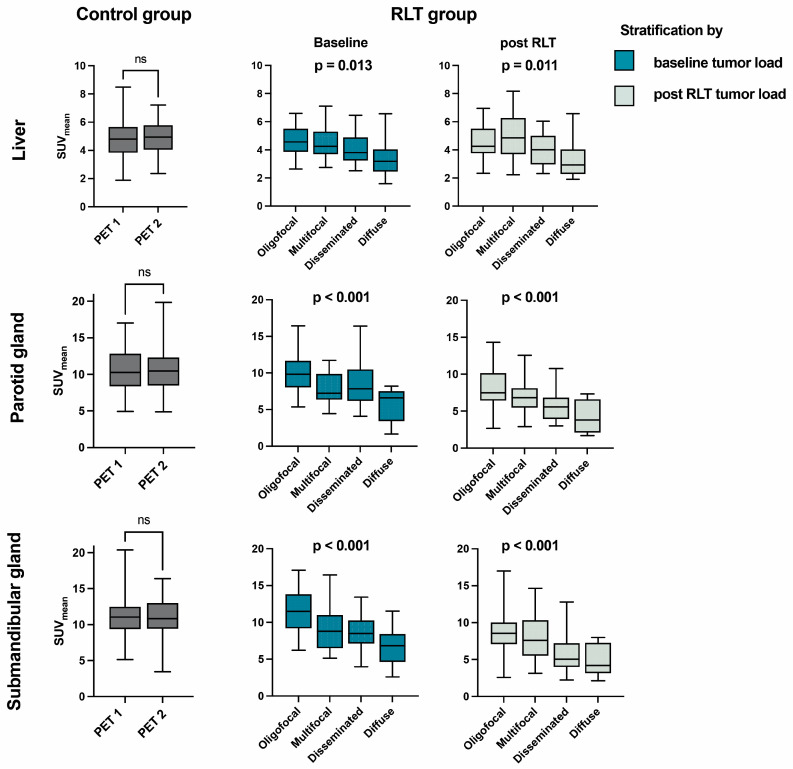
SUVmean of reference organs examined in the control and RLT group. The RLT group is stratified by tumor burden class at baseline (blue) or after RLT (green). PET 1: first acquisition; PET 2: second acquisition; RLT: radioligand therapy; SUV: standardized uptake value, ns: not significant (*p* > 0.05).

In the control group (PET 1), SUV_mean_ values of the liver, parotid and submandibular glands were 4.9 ± 1.4, 10.8 ± 3.2 and 11.3 ± 2.8; SUV_max_ values were 7.7 ± 2.0, 16.8 ± 5.4 and 18.1 ± 4.7. The relation of parotid to liver tissue uptake was thus 2-fold. Intrapatient comparison of the two acquisitions within the control group (PET 1 and PET 2) showed no significant differences in uptake to the liver (*p* = 0.920), parotid (*p* = 0.981) or submandibular gland (*p* = 0.602) (Table 2, Figure 1). 

Upon intergroup comparison, the oligometastatic subgroup yielded no significant decrease in uptake to any of the reference organs when compared to controls at baseline, while in groups with higher baseline tumor burden, i.e., the multifocal, disseminated, and diffuse group, significantly lower baseline values became increasingly apparent for salivary glands (−27.1% in multifocal to −55.8% parotid uptake in diffuse tumor burden) and the liver (−30.4% in diffuse tumor burden), as further detailed in Table 3. Subgroup comparison in the RLT group showed a significant decrease of uptake to the liver, parotid and salivary gland, with increasing tumor burden class (*p* = 0.013, *p* < 0.001, *p* < 0.001). This sink effect to non-tumoral tissue was more marked in salivary glands, as also reflected by a decreasing ratio of parotid to liver uptake. 

**Figure 2:**
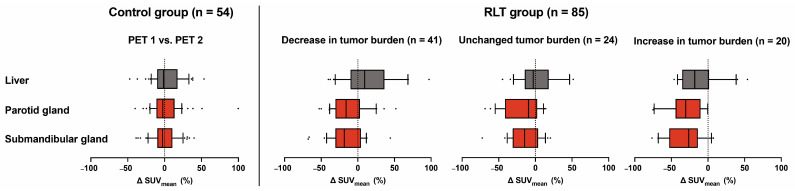
Standardized uptake values (SUV_mean_) in the liver (gray) and salivary glands (red) remain stable in the control group when comparing the first (PET 1) and second PET/CT acquisition (PET 2). In the RLT group, a significant decrease in both parotid and salivary glands is seen in all subgroups analyzed. Liver uptake is inversely associated with tumor burden change.

**Figure 3:**
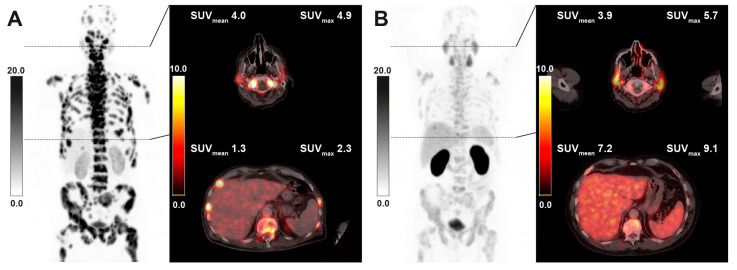
Sample [^68^Ga]Ga-PSMA-11 PET/CT images of a 65-year-old patient with diffuse bone marrow involvement receiving 6 cycles of RLT with cumulative 53.5 GBq of [^177^Lu]Lu-PSMA-617. Maximum intensity projection (MIP) images (left) with axial fusion images showing the parotid gland and liver uptake at baseline (**A**) and after RLT (**B**). The decrease in tumor burden is associated with markedly increasing liver uptake, while salivary uptake remains nearly unchanged.

**Figure 4:**
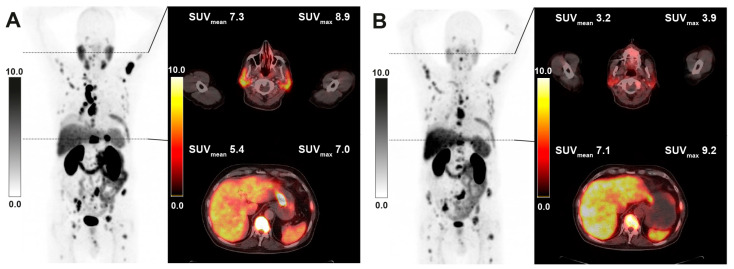
Sample [^68^Ga]Ga-PSMA-11 PET/CT images of a 64-year-old patient with multifocal metastatic disease receiving 2 cycles of RLT with cumulative 13.9 GBq of [^177^Lu]Lu-PSMA-617. Maximum intensity projection (MIP) images (left) with axial fusion images showing the parotid gland and liver uptake at baseline (**A**) and after RLT (**B**).

In the analysis of intraindividual changes after RLT, lower uptake values were seen in both parotid (−15.6%, *p* < 0.001) and salivary glands (−18.8%, *p* < 0.001) overall, while uptake in the liver was not significantly changed for the whole cohort (+7.2%, *p* = 0.212) (Table 4, Figure 2). In the subgroup of 41 patients with decreasing tumor burden under RLT, liver uptake showed an increase (+18.6%, *p* = 0.020), whilst in this group, parotid and submandibular uptake also significantly decreased compared to pretreatment values (−9.4%, *p* < 0.001 and −10.4%, *p* = 0.004, respectively). The inverse relation between tumor burden class and uptake parameters in group comparison was maintained in all reference organs when stratifying post RLT PET scans by respective tumor burden classification upon image acquisition (Figure 1). A sample image of a patient with markedly decreasing tumor burden and increasing liver uptake after RLT is provided in Figure 3. The diverging change in liver and salivary gland tissue uptake after RLT is shown in Figure 4. Clinically relevant changes to liver function parameters or therapy-related adverse events over the course of RLT were not observed (Table 5).

**Figure 5:**
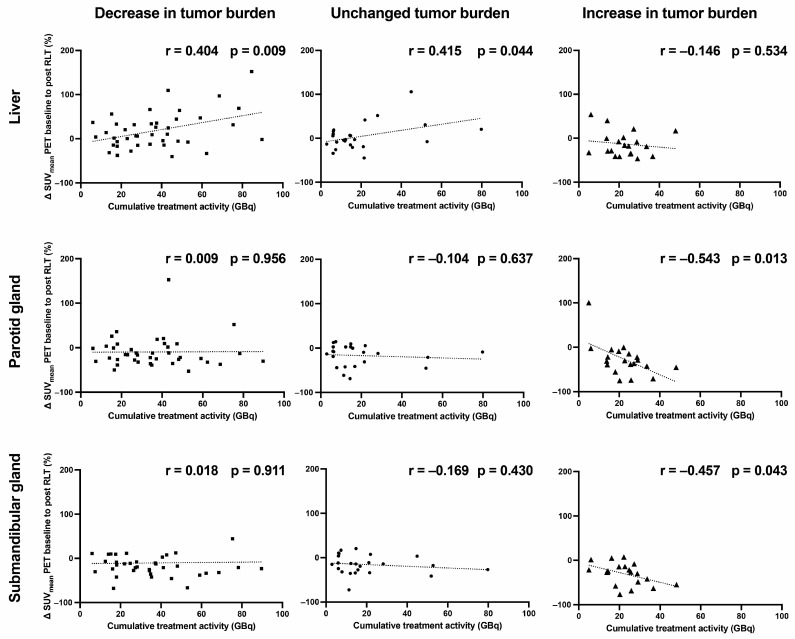
SUV_mean_ change (%) in reference organs under radioligand therapy in relation to cumulative treatment activity. SUV: standardized uptake value; GBq: gigabecquerel.

Cumulative treatment activity over the course of RLT in relation to uptake change in liver, parotid and salivary gland tissue was examined in three subgroups: in patients with (1) decreasing (n = 41), (2) unchanged (n = 24), and (3) increasing tumor burden (n = 20). In the subgroup of RLT patients who had an increase in tumor burden, a significant decrease in salivary gland uptake values could be observed in correlation with cumulative treatment activity in both the parotid (r = −0.543, *p* = 0.013) and submandibular gland (r = −0.457, *p* = 0.043) (Figure 5). While the liver uptake showed a rising tendency for the groups of unchanged (r = 0.415, *p* = 0.044) and decreasing (r = 0.404, *p* = 0.009) tumor burden, a corresponding tendency could not be seen for the salivary glands. 

## 4. Discussion

The change in uptake to reference organs on PET/CT imaging was assessed over the course of RLT in a sizable cohort of patients with mCRPC. Both at baseline and after RLT, higher tumor burden was associated with lower uptake in salivary gland and liver tissue. Throughout RLT, a significant decrease in salivary gland uptake was observed intraindividually regardless of the initial tumor burden and its respective intratherapeutic changes. 

Intraindividual changes in reference organ uptake were also assessed in a control group of patients with localized or low-volume disease and no major change in tumor burden over the course of 12 months. The baseline values found, compare with other studies in treatment-naïve patients with low-volume disease, the salivary glands showing approximately 2-fold higher SUV_mean_ values than healthy liver tissue [21,22]. In accordance with findings from test–retest studies, no relevant changes in uptake values could be seen between the two acquisitions [23,24]. 

Our study adds further evidence to the existence of a tumor burden-dependent “sink effect“ in salivary glands and, to a lesser extent, in healthy liver tissue. This phenomenon has been previously investigated in somewhat diverging reports [15,16,25,26]. Gaertner et al. conducted an analysis in a cohort visually stratified into 3 classes of tumor burden. They found a significant decrease in SUV_mean_ to parotid glands in 19 patients with medium (−31.4%) and 32 patients with high (−53.4%) tumor burden compared to 82 patients with low tumor burden; in liver tissue, only the high tumor burden class showed a significant decline in uptake (−18.3%) [25]. Werner et al. assessed 40 patients with predominantly low-volume disease and could not confirm a tumor sink in salivary glands or liver tissue in scans performed with [^18^F]F-DCFPyL [27]. The so-far largest static cohort, including 275 mCRPC and 81 hormone-sensitive prostate cancer patients, was investigated by Gafita et al. [15]. In their study, baseline PSMA-positive tumor burden was segmented by a dedicated quantification tool (qPSMA), stratified into five quintiles of tumor volume and compared to a control group with PSMA-negative scans (n = 50). In comparison to controls, significantly lower SUV_mean_ values were seen in salivary glands of patients with high to very high tumor volume, with −24.5 and −38.1%, respectively. A decrease in liver uptake was detected in all groups except the very low-volume group. These results are well in line with our findings, where decrease rates were non-significant in the oligometastatic group and showed significantly decreasing values in higher volume disease burden for the liver (−30.4% in diffuse tumor burden class), parotid (up to −55.8%) and submandibular glands (up to −45.6%).

To the best of our knowledge, only two studies have so far addressed the change in uptake to reference organs throughout RLT, both in comparatively small cohorts after a maximum of two RLT cycles. In the above-mentioned study by Gafita et al., a subgroup of 20 patients with very high-volume disease was re-examined after undergoing RLT. Results showed a tendency toward increased salivary gland (+61.1%, *p* = 0.06) and liver uptake (+33.4%, *p* = 0.17) in 10 responders, defined as patients with a ≥ 30% decline in PSMA-positive tumor volume after 2 RLT cycles. Non-responders (n = 10) had non-significant changes to salivary gland and liver uptake (−2.5% *p* = 0.67 and +12.1% *p* = 0.16). In a recent study conducted by Burgard et al. in 33 patients, 25 of which were responders to RLT, the post-therapeutic change in total PSMA-positive tumor volume (∆TLP) was inversely associated with uptake change (∆SUV_mean_) in salivary glands (r = −0.396, *p* = 0.023), with a non-significant analogous tendency in the liver (r = −0.300, *p* = 0.089). In the group of 25 responders to 2 cycles of RLT, a significant increase of parotid SUV_mean_ was shown (6.7 ± 2.1 to 7.6 ± 2.5, *p* = 0.022), while the liver uptake remained unchanged (*p* = 0.658). 

In our cohort with 85 treated patients, all patient groups (decreasing, unchanged, and increasing tumor burden) showed lower uptake values in salivary glands after RLT. A systematic rise with decreasing tumor burden was seemingly not observed per patient. This, at first sight contradictory finding to above studies, may be explained by the fact that in our cohort, patients were assessed after a longer course of RLT and not at an interim time point after only two treatment cycles. After multiple RLT cycles, PSMA expression on salivary gland tissue appears more markedly impacted. This result seems further supported by the observation that in patients with unchanged or decreasing tumor burden, liver and salivary glands showed diverging tendencies in relation to cumulative treatment activity. It can be hypothesized that in healthy liver tissue, a tumor sink/rise effect is maintained over time and follows intraindividual changes in tumor burden, while in salivary glands, the effect is outweighed by decreasing organ uptake through radiation effects of RLT. 

Salivary glands are known to be organs at risk during RLT with ^177^Lu- and ^225^Ac-based ligands [28]. Ligand accumulation is positively correlated to organ function, as shown by Li et al. [29]. Though it is understood that salivary glands exhibit PSMA expression to a significant extent, Rupp et al. found the tracer uptake in salivary glands not to be solely PSMA-mediated [30]. As a result, RLT with ^177^Lu-based ligands exposes salivary glands to radiation doses in the range of 0.4 to 0.8 Gy/GBq [31,32]. Therefore, the observed global decrease in salivary gland uptake might be a result of RLT-related tissue decline. Liver tissue is exposed to much lower doses during RLT whilst tolerant to higher levels of radiation. Okamoto et al. investigated mean liver dose after 4 RLT cycles, showing dose levels of 0.12 Gy/GBq [31]. In our cohort, no decline in liver function was observed, as reflected by unchanged liver enzymes (ALT, APT and GGT), as well as albumin and bilirubin levels. Changes in uptake after RLT are thus most likely predominantly attributable to tumor burden-associated effects.

In clinical routine, the introduction of 4-point visual scoring in addition to routine quantitative uptake values has been proposed by major lesion reporting guidelines, including E-PSMA and the recently updated PROMISE framework (v2) [12,13]. These scores allow for rapid, scanner-independent stratification of disease avidity by using the blood-pool, liver and salivary gland uptake as reference landmarks. Similar semiquantitative organ-derived scoring systems have been previously put in place for molecular imaging in neuroendocrine tumors (Krenning score) [33] and FDG-PET/CT response assessment in lymphomas (Deauville criteria) [34]. While a useful measure for baseline assessment, the role of such reference organ-derived scoring systems in mCRPC patients undergoing RLT remains under investigation [8]. With the apparent change in uptake to reference organs in absolute terms and in relation to tumor burden, the utility of reference organ-derived lesion metrics as response markers must be called into question. Furthermore, quantification of total PSMA-positive tumor volume on the basis of liver uptake-derived thresholding has gained importance for both prognostication and RLT response assessment, as introduced by RECIP criteria [35,36]. Here too, to avoid false findings, changes in liver uptake throughout RLT should be taken into consideration. 

The presence of tumor sink effects has also been investigated in the context of dosimetric studies. Violet et al. found higher tumor volume to result in lower intratherapeutic organ doses in both salivary glands and kidneys, which are both potential organs at risk [32]. Single-time-point PET/CT has limitations in accurately predicting intratherapeutic doses to non-target organs but may yet provide a suitable estimate of tracer distribution prior to RLT [37]. Future studies may take the tumor sink effect into account to provide a rationale for increased treatment activities in patients with extensive tumor burden [38,39]. 

Limitations of this study include its retrospective nature and limited population size, which may potentially have an impact on the strength of conclusions drawn in subgroup analyses. Second, in our study, only the widely established tracer [^68^Ga]Ga-PSMA-11 was examined. Results may differ for ^18^F-based tracers, also due to the effect of hepatic excretion. Third, our study relied on modified PROMISE criteria to visually stratify tumor burden, as readily applicable in routine assessment. Outside clinical routine, semiautomatic quantification tools of total PSMA-positive tumor load are under investigation and may play an increasingly important role in the future [9,40]. With the proposed volume detection algorithms relying on liver uptake-derived thresholds for segmentation, confounding effects for this analysis could not have been fully ruled out.

## 5. Conclusions

Uptake to liver and salivary gland tissue quantified by [^68^Ga]Ga-PSMA-11 PET/CT is inversely related to tumor burden prior to and following RLT with [^177^Lu]Lu-PSMA-617. Per patient, salivary gland uptake is further reduced throughout RLT independently from tumor burden, while changes in liver uptake remain burden-dependent effects. Liver and salivary gland uptake-derived metrics and segmentation thresholds may thus be of limited value when used as reference for response assessment to RLT.

## Figures and Tables

**Table 1:** Baseline characteristics of the examined groups. Data presented with median and interquartile range (IQR) or n (%). RLT: radioligand therapy; PSA: prostate-specific membrane antigen; mCRPC: metastatic castration-resistant prostate cancer. Subheadings are bolded.

Value	Control Group (n = 54)	RLT Group (n = 85)
Age	67 (60–73)	72 (65–77)
Body weight (kg)	85 (81–95)	82 (72–93)
PSA (µg/L)	0.8 (0.6–2.6)	64.0 (7.5–200.0)
**Total tumor burden at baseline**		
localized	33 (61)	0 (0)
oligofocal	21 (39)	28 (33)
multifocal	-	24 (28)
disseminated	-	20 (24)
diffuse	-	13 (15)
**Sites of metastases at baseline**		
Bone	11 (20)	74 (87)
Lymph node	18 (33)	75 (88)
Visceral	0 (0)	12 (14)
Bone + lymph node	1 (2)	65 (76)
Bone + lymph node + visceral	0 (0)	10 (12)
**Prior systemic therapies for mCRPC**		
Abiraterone	-	52 (61)
Enzalutamide	-	46 (54)
Radium-223-dichloride	-	20 (24)
Docetaxel	-	36 (42)
Cabazitaxel	-	16 (19)

**Table 2:** Paired intragroup comparison of uptake values in reference organs. Data presented with standard deviation. PET 1: first acquisition; PET 2: second acquisition; pre RLT PET: baseline PET/CT; post RLT PET: follow-up PET/CT after radioligand therapy.

Organ			Metric	Control Group (n = 54)			RLT Group (n = 85)		
				PET 1	PET 2	*p*	pre RLT PET	post RLT PET	*p*
Liver			SUV_mean_	4.9 ± 1.4	4.9 ± 1.2	0.920	4.3 ± 1.2	4.4 ± 1.4	0.424
			SUV_max_	7.7 ± 2.0	7.6 ± 1.8	0.715	6.7 ± 1.7	6.8 ± 1.9	0.810
Parotid gland			SUV_mean_	10.8 ± 3.2	10.7 ± 3.2	0.981	8.3 ± 3.3	6.8 ± 2.9	<0.001
			SUV_max_	16.8 ± 5.4	16.9 ± 5.6	0.899	12.7 ± 4.8	10.3 ± 4.3	<0.001
Submandibular gland			SUV_mean_	11.3 ± 2.8	11.1 ± 2.7	0.602	9.2 ± 3.4	7.3 ± 3.5	<0.001
			SUV_max_	18.1 ± 4.7	17.9 ± 4.4	0.633	14.2 ± 5.1	11.4 ± 5.2	<0.001

**Table 3:** Uptake values to reference organs in the RLT group, stratified by baseline tumor burden compared to the control group. Data presented as mean with standard deviation. CO: control; Ratio P/L: ratio of parotid to liver uptake. * PET1, ^†^ pre RLT PET.

Group	n	Liver			Parotid Gland			Submandibular Gland			Ratio P/L
		SUV_mean_	∆ (%) vs. CO	*p*	SUV_mean_	∆ (%) vs. CO	*p*	SUV_mean_	∆ (%) vs. CO	*p*	
Control group *	54	4.9 ± 1.4	-		10.7 ± 3.2			11.3 ± 2.8			2.2
RLT group ^†^											
-oligofocal	28	4.6 ± 1.0	−5.9	0.763	10.1 ± 3.1	−5.7	0.831	11.5 ± 3.0	1.8	0.996	2.2
-multifocal	24	4.5 ± 1.2	−6.7	0.704	7.8 ± 2.0	−27.1	<0.001	9.1 ± 2.8	−19.5	0.007	1.7
-disseminated	20	4.1 ± 1.1	−15.1	0.088	8.6 ± 3.3	−20.1	0.022	8.3 ± 2.5	−26.3	<0.001	2.1
-diffuse	13	3.4 ± 1.3	−30.4	0.001	4.7 ± 3.0	−55.8	<0.001	6.1 ± 3.1	−45.6	<0.001	1.4

**Table 4:** Intraindividual change of uptake values between the two PET acquisitions in both groups, with the RLT group stratified by change in tumor burden throughout RLT. Data presented as mean change (%) with standard deviation.

Group	n	Liver		Parotid Gland		Submandibular Gland	
		∆SUV_mean_ (%)	*p*	∆SUV_mean_ (%)	*p*	∆SUV_mean_ (%)	*p*
Control group	54	+3.7 ± 27.6	0.920	+2.3 ± 22.1	0.981	−0.1 ± 17.1	0.602
RLT group	85	+7.2 ± 37.6	0.212	−15.6 ± 33.3	<0.001	−18.8 ± 38.0	<0.001
-decrease in tumor burden	41	+18.6 ± 41.0	0.020	−9.4 ± 35.0	<0.001	−10.4 ± 46.3	0.004
-unchanged tumor burden	24	+4.5 ± 31.3	0.924	−16.7 ± 23.7	0.002	−15.5 ± 21.5	0.002
-increase in tumor burden	20	−13.1 ± 28.3	0.043	−26.9 ± 37.5	0.004	−30.4 ± 24.5	<0.001

**Table 5:** Blood parameters of patients in the RLT group with mean intraindividual change from baseline. AST: aspartate aminotransferase; ALT: alanine aminotransferase; GGT: gamma-glutamyl transferase; RLT: radioligand therapy; CTCAE: Common Terminology Criteria of Adverse Events.

	Albumin (g/dL)	Bilirubin (mg/dL)	AST (U/L)	ALT (U/L)	GGT (U/L)
Baseline (mean ± SD)	4.3 ± 0.4	0.4 ± 0.2	32 ± 22	22 ± 23	33 ± 25
post RLT (mean ± SD)	4.1 ± 0.4	0.4 ± 0.2	36 ± 28	22 ± 18	41 ± 38
intraindividual change (mean ± SD)	−0.1 ± 0.4	0.0 ± 0.2	4 ± 20	0 ± 13	6 ± 37
intraindividual change (range)	−1.3–1.2	−0.6–0.4	−100–152	−57–40	−89–242
CTCAE grade (1/2/3/4)	1/0/0/0	0/0/0/0	11/2/0/0	3/1/0/0	4/2/0/0

## Data Availability

The datasets analyzed and/or analyzed during the current study are available from the corresponding author upon reasonable request.

## Supplementary Materials

The following are available online at https://www.mdpi.com/article/10.3390/cancers15153878/s1, Supplement S1: Side comparison of salivary gland uptake values. PET 1: first acquisition, PET 2: second acquisition, pre RLT PET: baseline PET/CT, post RLT PET: follow-up PET/CT after radioligand therapy.
