# Supplementary material for: Impact of [177Lu]Lu-PSMA-617 Radioligand Therapy on Reference Organ Uptake Assessed by [68Ga]Ga-PSMA-11-PET/CT
Source: Cancers (Basel). 2023 Jul 30;15(15):3878. doi: 10.3390/cancers15153878 (PMC10417367; doi:10.3390/cancers15153878)
Supplement: Supplementary file 1 [file cancers-15-03878-s001.zip › cancers-2526452-supplementary.pdf]

cancers MDPI

Supplement S1 Side comparison of salivary gland uptake values. PET 1: first acquisition, PET 2: second acquisition, pre RLT PET: baseline PET/CT, post RLT PET: follow-up PET/CT after radioligand therapy.

Group	PET	Metric	Parotid gland			Submandibular gland		
			right	left	p	right	left	p
**Control**	PET 1	SUV_{mean}	10.7±3.2	10.8±3.2	0.737	11.3±2.8	11.2±2.8	0.890
		SUV_{max}	16.8±5.4	17.0±5.2	0.654	18.1±4.7	18.0±4.5	0.704
	PET 2	SUV_{mean}	10.7±3.2	11.1±3.3	0.138	11.1±2.7	11.2±2.8	0.666
		SUV_{max}	16.9±5.6	17.6±5.4	0.145	17.9±4.4	18.0±4.5	0.716
**RLT group**	pre RLT PET	SUV_{mean}	8.3±3.3	8.4±3.6	0.536	9.2±3.4	8.9±3.3	0.106
		SUV_{max}	12.7±4.8	13.0±5.1	0.271	14.1±5.1	13.8±4.8	0.177
	post RLT PET	SUV_{mean}	6.8±2.9	6.6±3.1	0.442	7.4±3.3	7.2±3.3	0.188
		SUV_{max}	10.3±4.3	10.2±4.2	0.565	11.4±5.2	11.0±4.8	0.061
